# Supplementary material for: Number of Teeth Is Related to Craniofacial Morphology in Humans
Source: Biology (Basel). 2022 Apr 1;11(4):544. doi: 10.3390/biology11040544 (PMC9029740; doi:10.3390/biology11040544)
Supplement: Supplementary file 1 [file biology-11-00544-s001.zip › biology-1625219-supplementary.pdf]

Number of teeth is related to craniofacial shape in humans

Elias S. Oeschger, Georgios Kanavakis, Alina Cocos, Demetrios J. Halazonetis, Nikolaos Gkantidis

Table S1. Multivariate regression of shape on age and number of missing teeth, including third molars, in female (N=476) and male (N=332) samples. Each shape configuration was described with the number of PCs explaining more than 85% of shape variation, as assessed with the broken-stick method.

Shape configurations			η^2	P-value
Cranial Base (PC1-PC9)	Females	Age	0.122	<0.001*
		Number of missing teeth	0.035	0.056
	Males	Age	0.123	<0.001*
		Number of missing teeth	0.034	0.253
Maxilla (PC1-PC10)	Females	Age	0.142	<0.001*
		Number of missing teeth	0.075	0.001*
	Males	Age	0.198	<0.001*
		Number of missing teeth	0.157	<0.001*
Mandible (PC1-PC8)	Females	Age	0.160	<0.001*
		Number of missing teeth	0.061	<0.001*
	Males	Age	0.233	<0.001*
		Number of missing teeth	0.098	<0.001*
Entire craniofacial configuration (PC1-PC18)	Females	Age	0.371	<0.001*
		Number of missing teeth	0.188	<0.001*
	Males	Age	0.443	<0.001*
		Number of missing teeth	0.205	<0.001*

*P<0.05

Table S2. Multivariate regression of shape (without allometry) on age and number of missing teeth, including third molars, in female (N=476) and male (N=332) samples. Each shape configuration was described with the number of PCs explaining more than 85% of shape variation, as assessed with the broken-stick method.

Shape configurations			η^2	P-value
Cranial Base (PC1-PC9)	Females	Age	0.000	1.000
		Number of missing teeth	0.034	0.066
	Males	Age	0.000	1.000
		Number of missing teeth	0.036	0.215
Maxilla (PC1-PC10)	Females	Age	0.000	1.000
		Number of missing teeth	0.062	<0.001*
	Males	Age	0.000	1.000
		Number of missing teeth	0.138	<0.001*
Mandible (PC1-PC8)	Females	Age	0.000	1.000
		Number of missing teeth	0.079	<0.001*
	Males	Age	0.000	1.000
		Number of missing teeth	0.105	<0.001*
Entire craniofacial configuration (PC1-PC18)	Females	Age	0.000	1.000
		Number of missing teeth	0.175	<0.001*
	Males	Age	0.000	1.000
		Number of missing teeth	0.199	<0.001*

*P<0.05

Table S3. Multivariate regression of maxillary and mandibular shape on sex, age, and number of missing teeth (without considering third molars), performed on two subsamples that had no tooth agenesis and agenesis only in the maxilla or only in the mandible. Each shape configuration was described with the number of PCs explaining more than 85% of shape variation, as assessed with the broken-stick method.

Shape configurations		η^2	P-value
Individuals with tooth agenesis only in the maxilla (N=131) or no agenesis (n = 404)			
Maxilla (PC1-PC10)	Sex	0.045	0.006*
	Age	0.158	<0.001*
	Number of missing teeth	0.115	<0.001*
Mandible (PC1-PC8)	Sex	0.033	0.023*
	Age	0.179	<0.001*
	Number of missing teeth	0.017	0.323
Individuals with tooth agenesis only in the mandible (N=166) or no agenesis (n = 404)			
Maxilla (PC1-PC10)	Sex	0.042	0.007*
	Age	0.176	<0.001*
	Number of missing teeth	0.027	0.125
Mandible (PC1-PC8)	Sex	0.011	0.631
	Age	0.191	<0.001*
	Number of missing teeth	0.024	0.087

*P<0.05

Table S4. Multivariate regression of maxillary and mandibular shape on sex, age, and number of missing teeth (considering also third molars), performed on two subsamples that had no tooth agenesis and agenesis only in the maxilla or only in the mandible. Each shape configuration was described with the number of PCs explaining more than 85% of shape variation, as assessed with the broken-stick method.

Shape configurations		η^2	P-value
Individuals with tooth agenesis only in the maxilla (N=109) or no agenesis (n = 315)			
Maxilla (PC1-PC10)	Sex	0.042	0.062
	Age	0.147	<0.001*
	Number of missing teeth	0.083	<0.001*
Mandible (PC1-PC8)	Sex	0.025	0.240
	Age	0.189	<0.001*
	Number of missing teeth	0.021	0.353
Individuals with tooth agenesis only in the mandible (N=117) or no agenesis (n = 315)			
Maxilla (PC1-PC10)	Sex	0.036	0.116
	Age	0.173	<0.001*
	Number of missing teeth	0.048	0.022*
Mandible (PC1-PC8)	Sex	0.024	0.242
	Age	0.205	<0.001*
	Number of missing teeth	0.032	0.091

*P<0.05

(**a**)

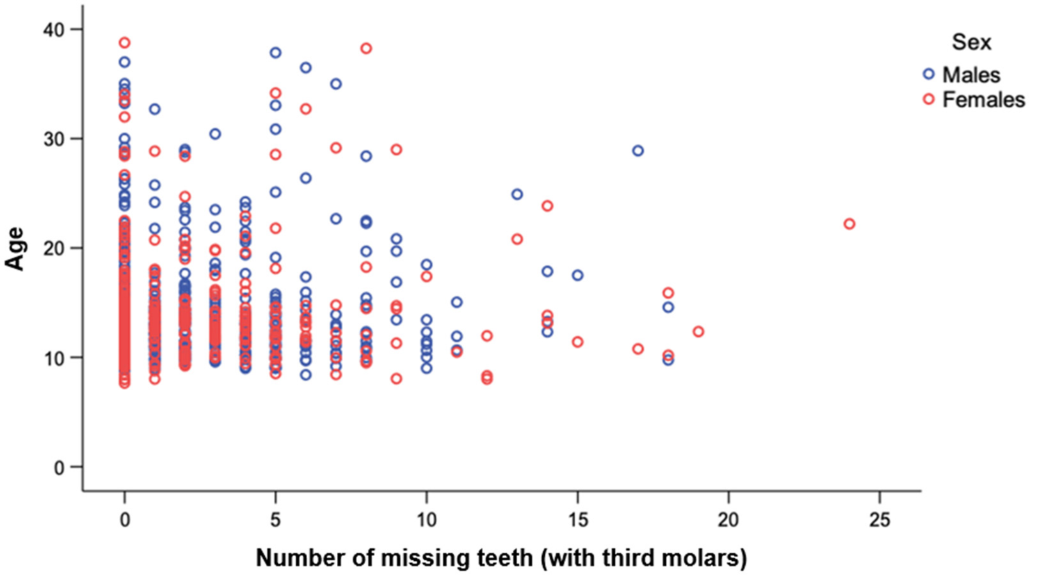

(**b**)

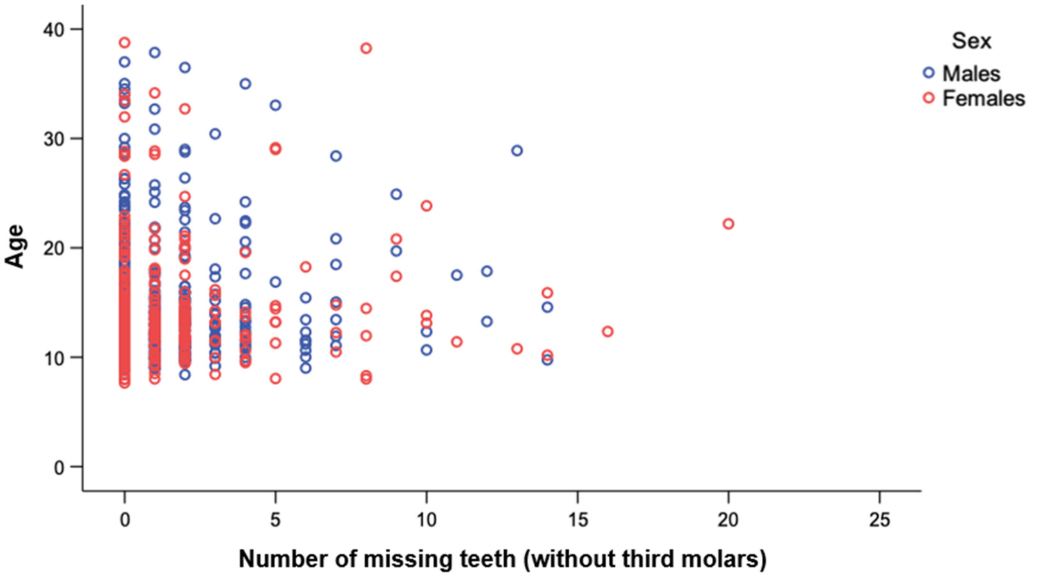

Figure S1. Sample distribution according to age, sex, and number of missing third molars (a) with and (b) without considering third molars.

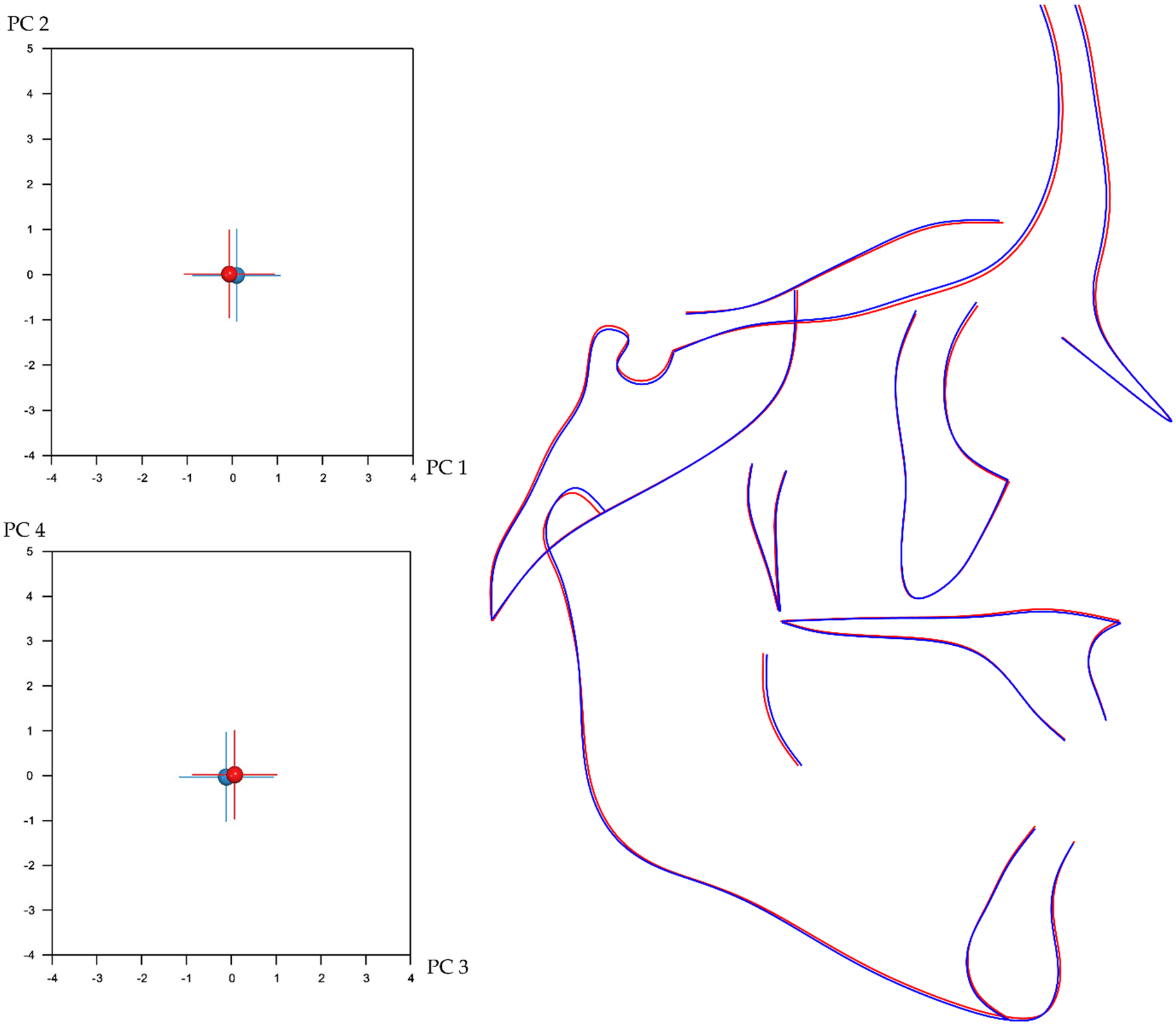

Figure S2. Left: Average difference between males (blue) and females (light red) in entire cranial configuration shape as explained by PC1 (21.8%) – PC2 (15.5%) (top) and PC3 (7.8%) – PC4 (6.2%) (bottom) after removal of the allometry effect in the sample. The numbers in the parentheses represent the percentage of variation explained by each PC. Right: Best fit superimposition of average male (blue) and average female (light red) craniofacial configurations.

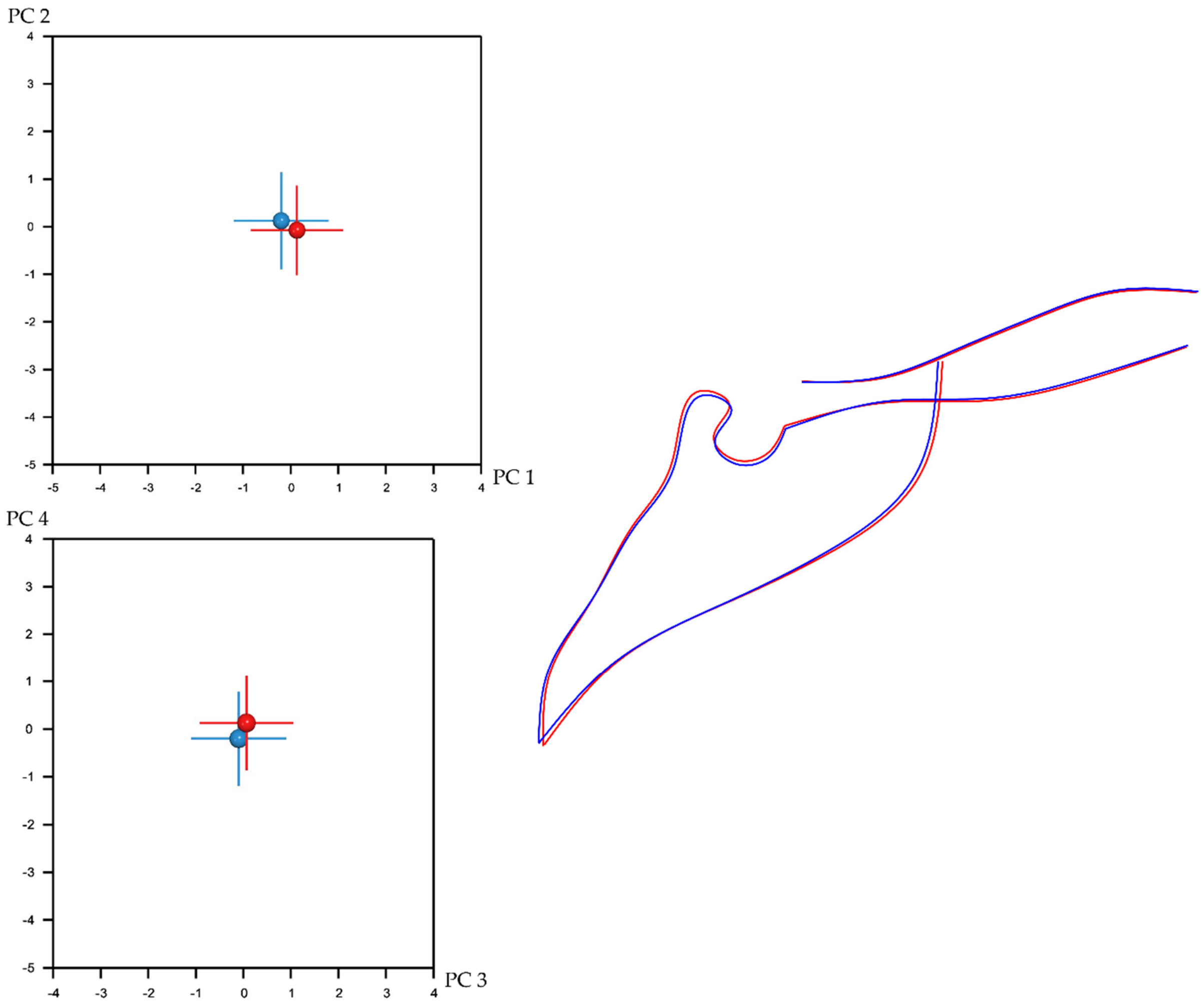

Figure S3. Left: Average difference between males (blue) and females (light red) in cranial base shape as explained by PC1 (17.4%) – PC2 (16%) (top) and PC3 (11.2%) – PC4 (9.8%) (bottom) after removal of the allometry effect in the sample. The numbers in the parentheses represent the percentage of variation explained by each PC. Right: Best fit superimposition of average male (blue) and average female (light red) cranial base configurations.

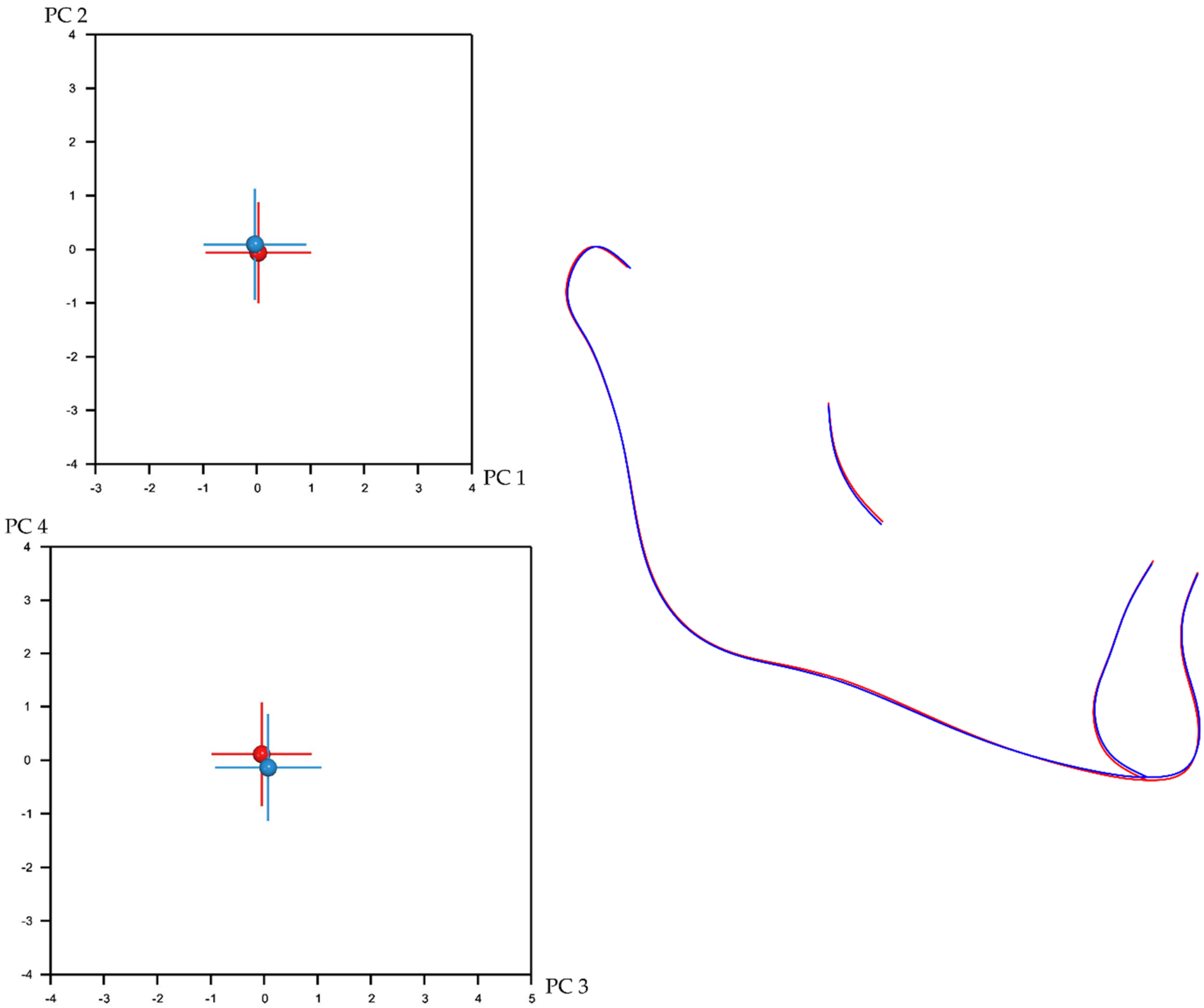

Figure S4. Left: Average difference between males (blue) and females (light red) in mandibular shape as explained by PC1 (24.9%) – PC2 (21.4%) (top) and PC3 (13.9%) – PC4 (11.3%) (bottom) after removal of the allometry effect in the sample. The numbers in the parentheses represent the percentage of variation explained by each PC. Right: Best fit superimposition of average male (blue) and average female (light red) mandibular configurations.

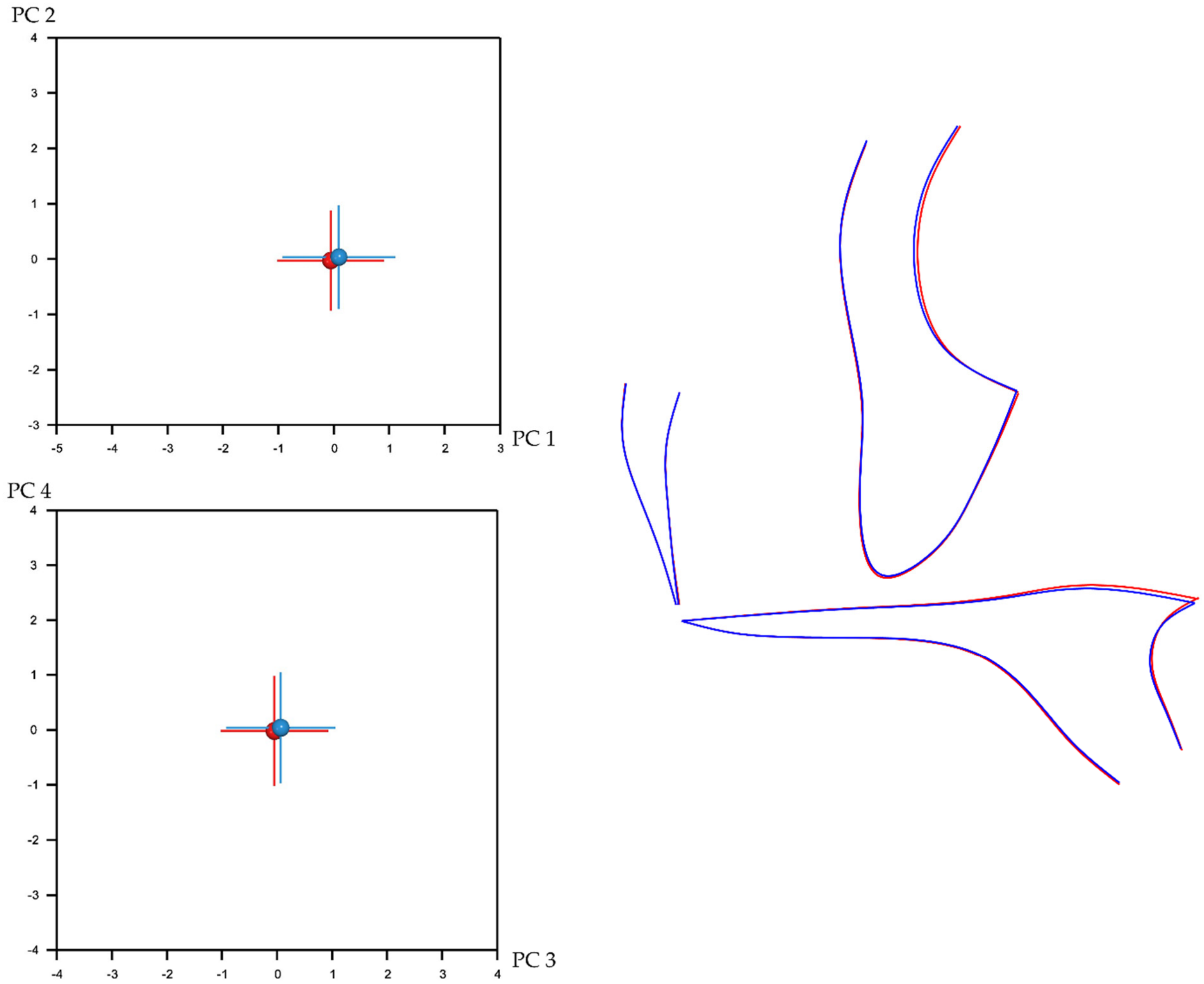

Figure S5. Left: Average difference between males (blue) and females (light red) in maxillary shape as explained by PC1 (21.3%)- PC2 (15.6%) (top) and PC3 (11.1%) – PC4 (7.7%) (bottom) after removal of the allometry effect in the sample. The number in the parentheses represent the percentage of variation explained by each PC. Right: Best fit superimposition of average male (blue) and average female (light red) maxillary configurations.

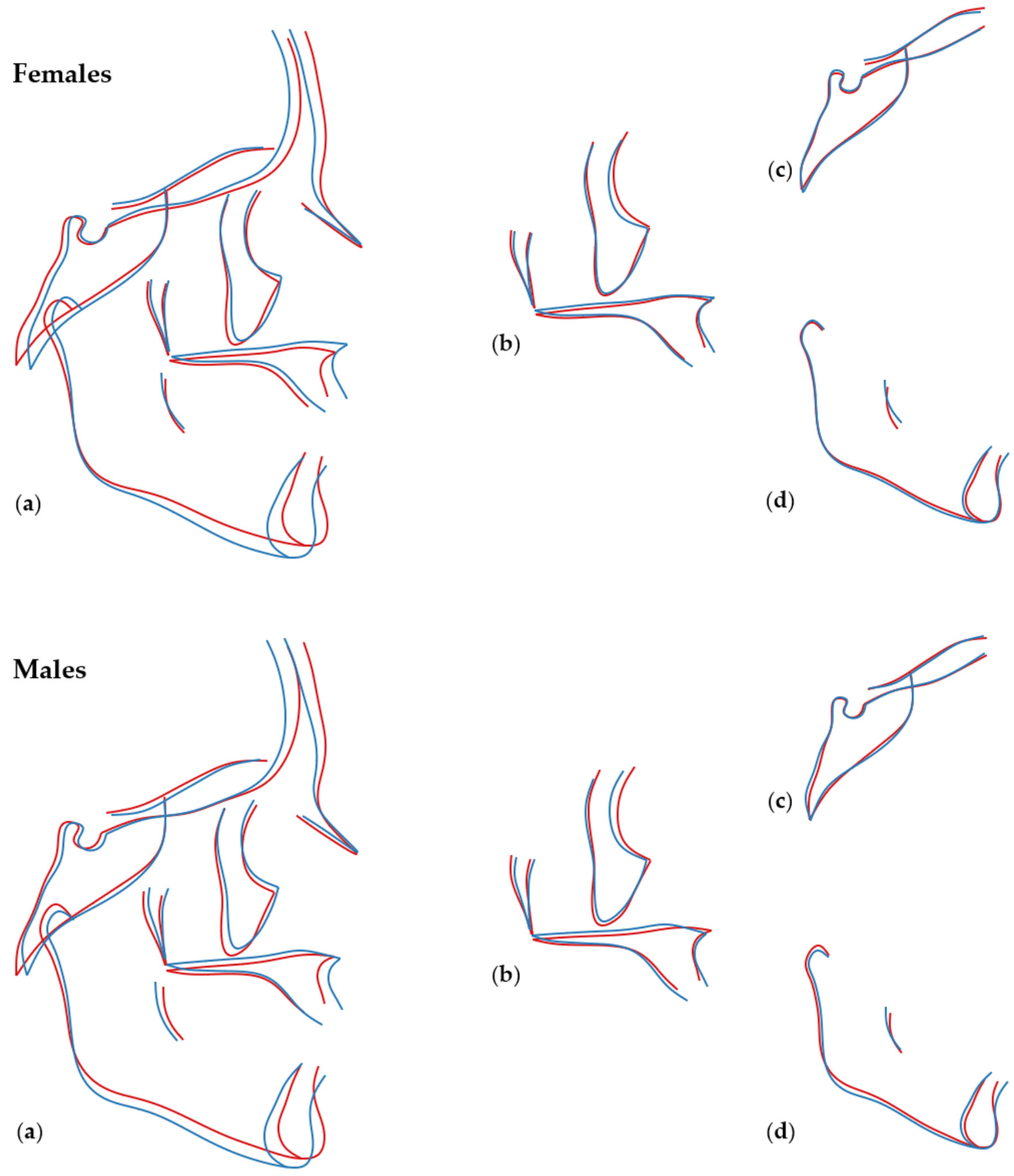

Figure S6. Regression of shape on number of missing teeth, including third molars, in females (top row) and males (bottom row). The blue lines represent shape configurations in cases with no or minimum missing teeth (-4SD from average shape), and the red lines represent shape configurations in cases with maximum number of missing teeth (+4SD from average shape). The following shape configurations are displayed: (a) entire cranial shape, (b) maxilla, (c) cranial base, and (d) mandible. Cranial base differences were not statistically significant.

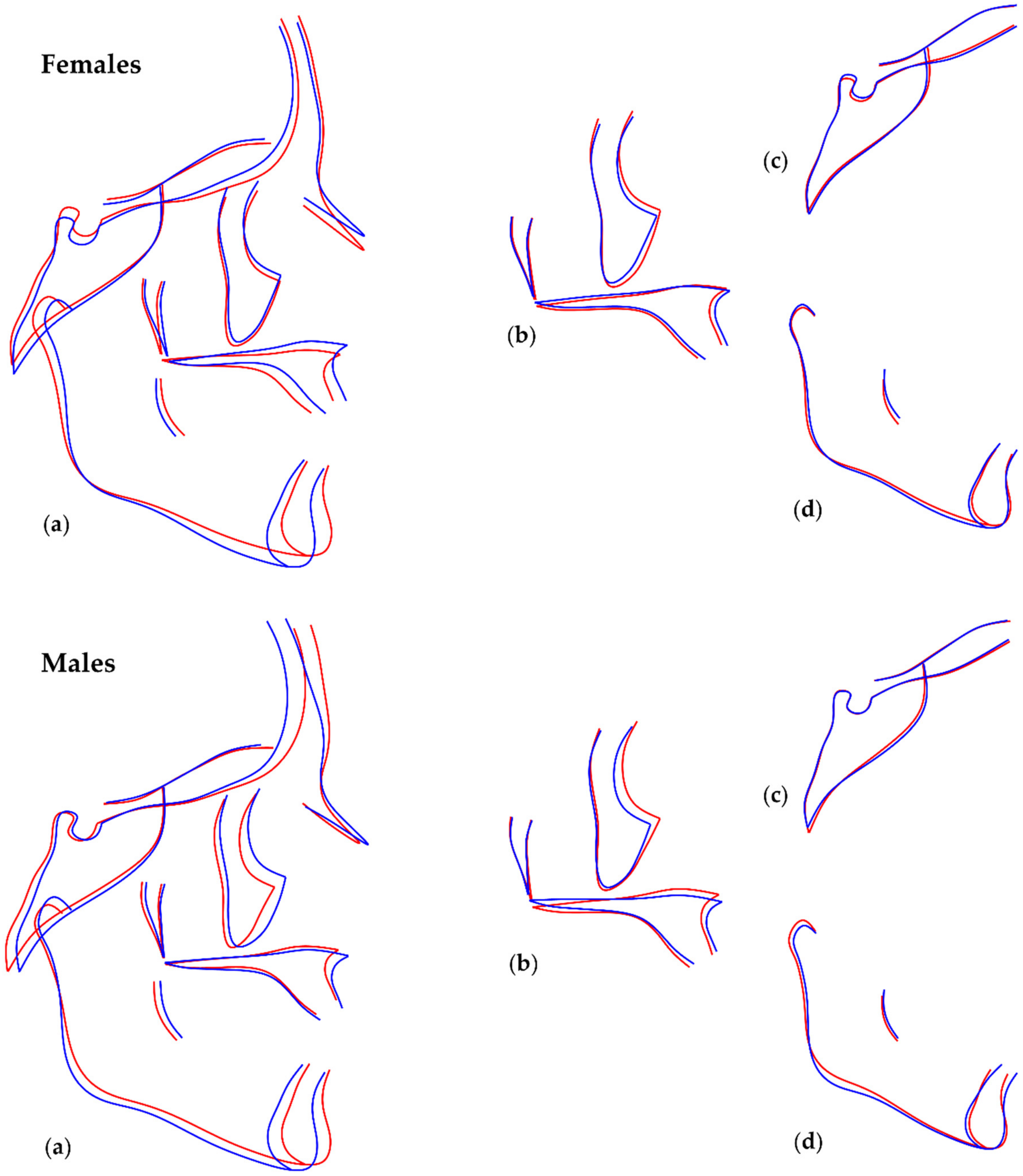

Figure S7. Regression of shape (after removal of allometry effect) on number of missing teeth, including third molars, in females (top row) and males (bottom row). The blue lines represent shape configurations in cases with no or minimum missing teeth (-4SD from average shape), and the red lines represent shape configurations in cases with maximum number of missing teeth (+4SD from average shape). The following shape configurations are displayed: (a) entire cranial shape, (b) maxilla, (c) cranial base, (d) mandible. Cranial base differences were not statistically significant.
